# Could “Low Quality of Life” Rather Than “Significant Symptoms” Be Better Criteria for the Selection of Patients for the Repair of a Giant Hiatus Hernia?

**DOI:** 10.7759/cureus.55740

**Published:** 2024-03-07

**Authors:** Mohammed Elniel, Madhu Chaudhury, Nasreen Desai, Christina Lo, Ravindra S Daté

**Affiliations:** 1 General Surgery, Lancashire Teaching Hospitals, Preston, GBR

**Keywords:** hiatus hernia repair, qolrad, laparoscopic surgery, giant hiatus hernia, quality of life

## Abstract

Purpose

Giant hiatus hernia (GHH) repair is undertaken electively in symptomatic patients, to prevent complications such as gastric volvulus and subsequent mortality. Advances in laparoscopy and perioperative care have reduced the risk of GHH repair, and improvement in quality of life (QoL) has become an important outcome measure. In our unit, we have been assessing QoL in all the operated as well as non-operated patients with GHH, using the “Quality of Life in Reflux and Dyspepsia” (QOLRAD) questionnaire.

We sought to evaluate differences in QoL between patients who were managed conservatively for GHH and those who underwent GHH repair over a two-year period.

Methods

All patients seen in the Upper Gastrointestinal Tertiary Unit in Lancashire Teaching Hospitals NHS Trust with GHH between January 2015 and December 2022 were identified from a prospectively kept database. QOLRAD scores were analyzed and compared between conservatively and operatively managed patients using the Mann-Whitney U test. Demographic and operative outcome data were also collected.

Results

Eighty-seven patients with GHH were included. QoL of 51 patients improved significantly after elective surgery. Five out of 36 patients, who were initially treated conservatively, elected to have repair during their follow-up period. These 5 Patients had a lower initial QOLRAD score in comparison to those whose management remained conservative (2.72 vs 5.05, Mann Whitney U test p=0.034), and their QOLRAD scores also improved significantly after the operation. QOLRAD scores in conservatively managed patients remained stable over a two-year follow-up period.

Conclusion

Objectively calculated low QoL may be a more useful tool than subjective symptoms in selecting patients for elective repair of GHH.

## Introduction

Giant hiatus hernias (GHHs) are defined as the herniation of more than 30% of the stomach with or without abdominal viscera through the diaphragmatic esophageal hiatus [1,2]. The incidence of GHH rises with age, as such, patients presenting with GHH are often older with multiple co-morbidities [3]. Co-morbid patients with minimal symptoms are often managed conservatively. A Markov analysis by Stylopoulos in 2002 reported a low rate of annual risk of complication (1.1%) from GHH through “watchful waiting” management in comparison to the rate of mortality (1.4%) from elective repair [4].

However, mortality rates after elective repair of GHH are demonstrably lower than emergency repair (0.37% vs 5%-17%). Hence, the literature supports preventing complications of GHH and avoidance of emergency surgery as an indication for elective surgery [5,6]. In recent years, improvement in quality of life (QoL) post-GHH repair has been reported in the literature as an indication for surgery [7-10].

Morrow et al. demonstrated an improvement of 1.3 additional quality-adjusted life years (QALY) in patients undergoing elective hiatal hernia repair over those managed conservatively using hypothetical computer modeling [11]. SAGES guidance on hiatal hernia management cites symptomatology and acute gastric volvulus as indications for the repair of hernias [12]. Differences in QoL of operated and non-operated patients have not been evaluated before.

The Quality of Life in Reflux and Dyspepsia questionnaire (QOLRAD), amongst other QoL tools such as the Reflux Disease Questionnaire (DSQ), Short-Form 36 Item Health Survey (SF-36) and the Gastroesophageal Reflux Disease Health-Related Quality of Life metric (GERD-HRQL) has been used to evaluate QoL in specific groups undergoing hiatal hernia repair such as the elderly [7-10] or those undergoing hiatal hernia repair with mesh [13]. In our unit, QoL assessment with QOLRAD forms part of the workup for all patients with GHH.

In this observational study, we herewith present our prospectively kept data of all the patients presented to us with GHH. We also aim to analyze differences in QoL of operated and non-operated patients and seek to find its practical significance.

## Materials and methods

All patients who were referred to our unit (Upper Gastrointestinal Tertiary Unit in Lancashire Teaching Hospitals NHS Trust) between January 2015 and December 2022 with GHH, where GHH was defined as more than 30% of the stomach in the chest were identified from a prospectively kept database. All the patients completed QOLRAD questionnaires at their initial consultation. Based on SAGES guidelines, asymptomatic patients were advised to not undergo an operation at their initial consultation. A “watchful wait” approach was taken in these patients and were given further questionnaires at six months, 12 months, and 24 months for their postal follow-up. All the operated patients were given further questionnaires at six weeks, six months, 12 months, and 24 months postoperatively. Approval for the collection of this data to objectively assess any change in QoL was gained from our Research and Innovation department under the category of “Service Evaluation.”

For ease of clarification, patients who elected to have a repair of their GHH at the initial consultation will be referred to as the immediate operative group (IOG). Patients who were managed conservatively with no intention to undergo operative management shall be referred to as the non-operative group (NOG). Patients who were initially managed conservatively, but later opted to undergo elective GHH repair will be referred to as the delayed operative group (DOG).

Operative technique

**Figure 1:**
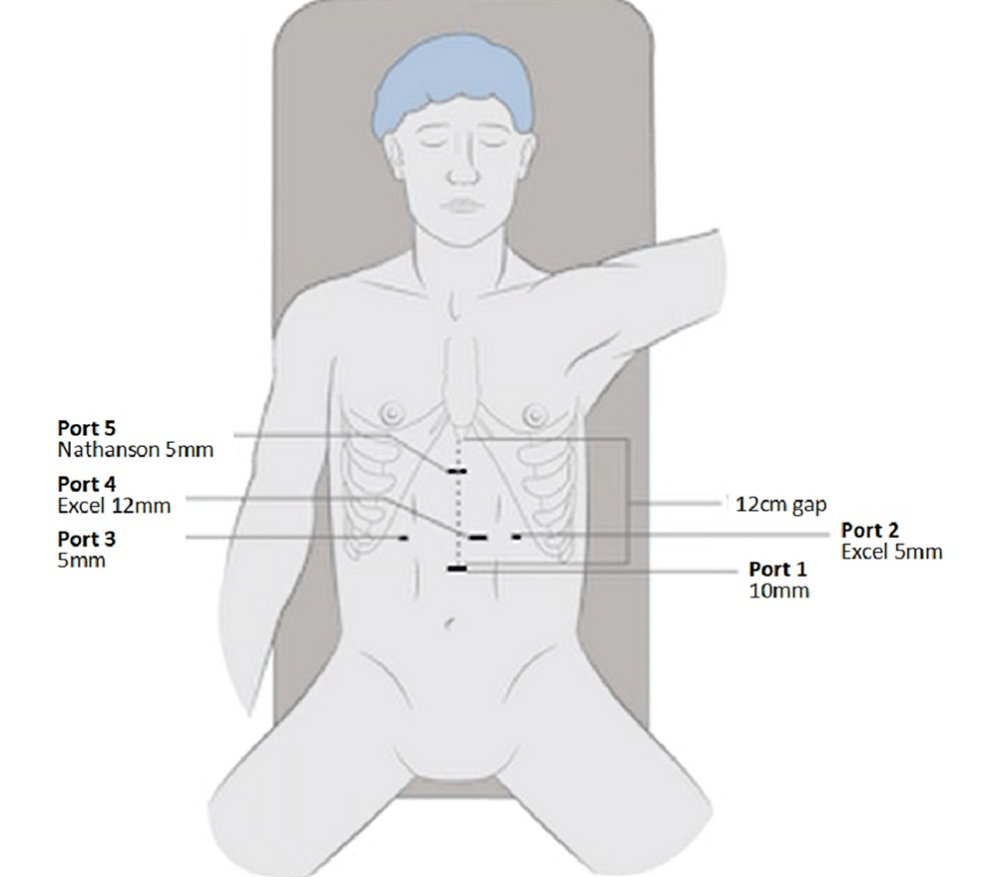
Schematic diagram of the port placement used in laparoscopic GHH repair Courtesy: Authors

All procedures were performed by the last author (RSD) or by his trainees under his direct supervision with two assistants. Patients were placed in a low lithotomy position. Repair of GHH is performed laparoscopically, through five small incisions. The 10 mm camera port is inserted in the midline approximately 14 cm below the xiphisternum. A 5-mm left subcostal port is inserted in the anterior axillary line, and a 12-mm port is inserted in the same transverse line, midway between the camera and the left subcostal port. a fourth right subcostal port is inserted in the anterior axillary line. A Nathanson retractor is placed 1 cm below the xiphisternum using a 5-mm trocar to create an entry into the peritoneum. This port placement is demonstrated in Figure 1. Variation in port placement is rarely required unless an enlarged left lobe of the liver or intra-abdominal adhesions preclude the standard port placement.

 

The hernial sac was identified and dissected away from the mediastinal structures to ensure adequate esophageal mobilization and reduction of the herniated stomach into the abdominal cavity, following which the sac was excised. Division of short gastric arteries and crural repair was routinely performed in all the patients. Nissen’s fundoplication was performed in patients with pre-operative reflux symptoms. Three-point fixation gastropexy was performed in patients with predominant obstructive symptoms and no history of reflux. Antropexy was performed if mesentero-axial volvulus was present.

Questionnaires

QoL data were collected using the QOLRAD questionnaire. QOLRAD is a disease-specific validated questionnaire that was developed to assess the quality of life of patients suffering from reflux and dyspeptic symptoms [14,15]. The questionnaire is comprised of 25 items covering five clinically relevant domains: emotional distress, sleep disturbance, food and drink problems, physical/social functioning, and vitality [15]. A 7-point Likert response scale is used to assess the frequency or severity of the item described. A total score is calculated as a mean as are the sub-scores covering each domain. A high QOLRAD score indicates a better QoL. Permission was obtained from AstraZeneca for the usage of the QOLRAD questionnaire. The QOLRAD questionnaire is included in Appendix 1.

Statistical analysis

Statistical analysis was completed using the statistical package GraphPad Prism. Comparison of QOLRAD total scores between DOG, IOG, and NOG were made using the Mann-Whitney U test. Changes in QOLRAD scores over time were compared using the Kruskal-Wallis test. Two-way ANOVA was used to compare changes in QOLRAD score over time between groups. Select data points (age and QOLRAD scores) of patients who underwent immediate operative management were also compared with the conservatively managed group of patients.

## Results

**Figure 2:**
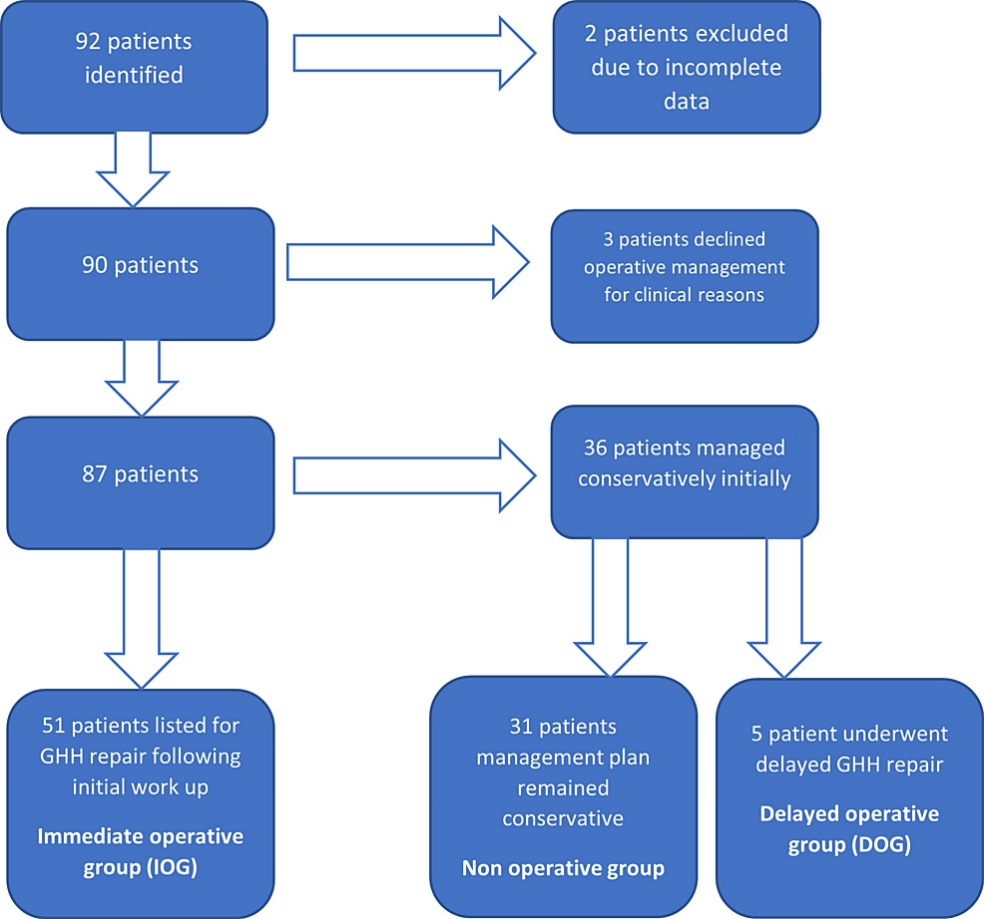
Patient classification flow chart This flow chart demonstrates the process of selecting eligible patients for participation in the study

Eighty-seven patients were included in the study. There were 51 patients in the IOG (median age 69.5). Of the 36 conservatively managed patients, five required elective GHH repair due to worsening symptoms, and the remaining 31 did not require operative management, hence there were 31 and five patients in the NOG and DOG, respectively (in whom the median ages were 74.0 and 66.2). Forty-one of the patients were female in the IOG, 22 in the NOG, and five in the DOG. The selection process is demonstrated by the flow chart in Figure 2. Patients who were declined GHH repair due to their medical co-morbidities were excluded.

One patient did not have crural repair, as their crura was deemed too friable for repair at the time of operation. Ten recurrences occurred across both groups. Four of these recurrences required a re-operation. Of these four, one was in the DOG. One patient in the IOG required a cardio-esophagectomy for a gastro-esophageal junction perforation in the early postoperative period, and one patient in the IOG required a re-do fundoplication for dysphagic symptoms, at the time of operation an injury to the fundus occurred necessitating a fundectomy. One patient in the NOG required an emergency GHH repair for hernia strangulation after their follow-up period had ended. There were no mortalities in this series. The operative data are summarized in Table 1.

**Table 1:** Summary of intraoperative events and postoperative outcomes between both operative groups

	IOG, N=51	DOG, N=5
Laparoscopic	100% (51)	100% (5)
Nissen’s fundoplication	72.5% (37)	60% (3)
Anterior 180 fundoplication	7.8% (4)	0% (0)
Posterior 180 fundoplication	2.0% (1)	0% (0)
No fundoplication	17.6% (9)	40% (2)
Gastropexy	70.6% (36)	40% (2)
Crural repair	98% (50)	100% (5)
Emergency	7.8% (4)	0% (0)
Recurrence	17.6% (9)	20% (1)
Re-operation	9.8% (5)	20% (1)
Median stay	2 days	2 days

QOLRAD scores

**Figure 3:**
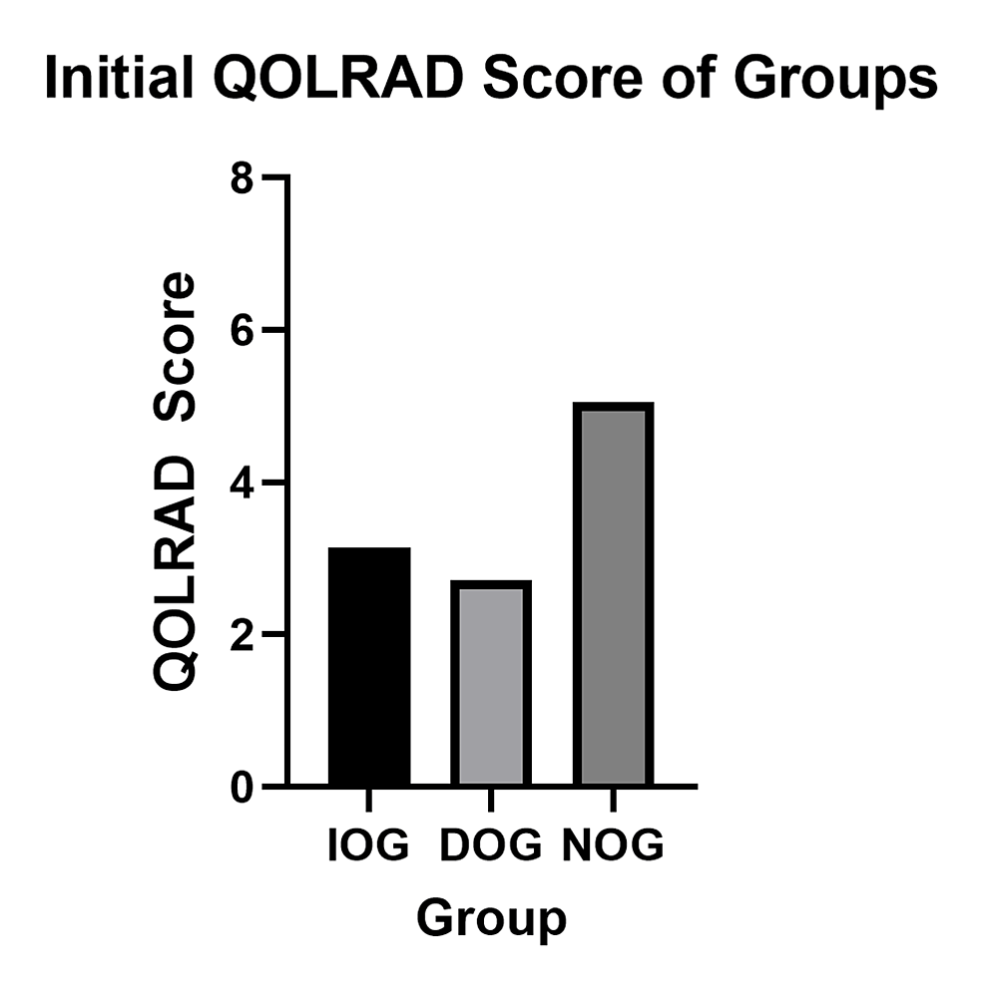
Overall QOLRAD score comparison between groups Overall initial mean QOLRAD scores of each group - IOG mean score: 3.15, DOG mean score: 2.72, NOG mean score: 5.05. Significant differences were found between IOG and NOG (p < 0.001) and DOG and NOG (p = 0.034), while no significant difference was observed between IOG and DOG (p = 0.743). QOLRAD - Quality of Life in Reflux and Dyspepsia

Fifty-five (55/87, 63%) of patients returned consecutive QOLRAD questionnaires (29/51, 60% in IOG, 5/5, 100% in DOG and 21/31, 67% in NOG). The mean overall initial QOLRAD scores of each group are demonstrated in Figure 3. Mann-Whitney U tests revealed significant differences in initial QOLRAD scores between IOG and NOG as well as between DOG and NOG (p<0.001 and p=0.034 respectively), whereas there was no significant difference between IOG and DOG (3.15 vs 2.72, p=0.743).

**Figure 4:**
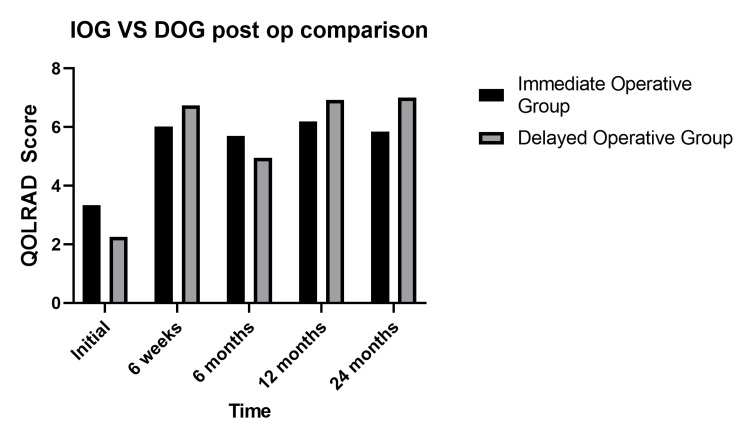
Comparison of QOLRAD scores following GHH repair between IOG and DOG Comparison of the mean scores between IOG and DOG following GHH repair over time. No significant difference was found between the two groups postoperatively (two-way ANOVA p=0.743). QOLRAD - Quality of Life in Reflux and Dyspepsia

Post GHH repair, patients in the DOG demonstrated significant improvement in their follow-up overall QOLRAD scores, which was comparable to the improvement seen in the IOG, demonstrated in Figure 4.

**Figure 5:**
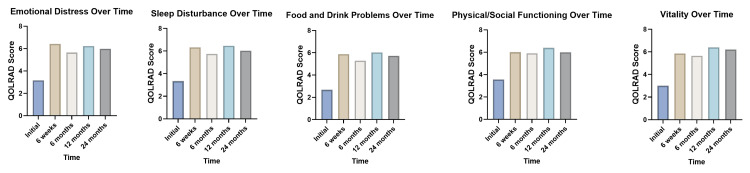
Column bar charts of QOLRAD scores in each domain over time postoperatively Column bar charts of mean QOLRAD scores of IOG and DOG over time in each domain postoperatively. Kruskal Wallis test demonstrated statistical significance between pre- and postoperative scores in all domains (p<0.001). QOLRAD - Quality of Life in Reflux and Dyspepsia

The combined QOLRAD scores of both IOG and DOG across all domains over the postoperative period are summarized in Table 2 and Figure 5. Statistically significant improvement in all QOLRAD domains postoperatively was observed when the data was analyzed using the Kruskal-Wallis test.

**Table 2:** Comparison table of QOLRAD scores pre- and postoperation QOLRAD questionnaire summary pre- and postoperation of IOG and DOG. Analysis completed using Kruskal-Wallis test.

QOLRAD Domain		Pre-op	6 weeks	6 months	12 months	24 months	P-value
Emotional distress	MEAN	3.2	6.4	5.6	6.2	6.0	<0.001
	95% CI	2.6 – 3.7	6.0 – 6.8	5.0 – 6.3	5.6 – 6.9	5.2 – 6.7	
	SD	1.7	0.8	1.8	1.7	1.6	
Sleep disturbance	MEAN	3.3	6.3	5.7	6.5	6.0	<0.001
	95% CI	2.8 – 3.9	5.9 – 6.8	5.0 – 6.5	6.0 – 6.9	5.2 – 6.9	
	SD	1.8	0.85	1.8	1.1	1.8	
Food and drink problems	MEAN	2.7	5.9	5.3	6.0	5.7	<0.001
	95% CI	2.1 – 3.3	5.2 – 6.6	4.6 – 6.0	5.4 – 6.6	4.9 – 6.5	
	SD	1.7	1.3	1.8	1.5	1.7	
Physical / social functioning	MEAN	3.6	6.0	5.9	6.4	6.0	<0.001
	95% CI	3.0 – 4.1	5.4 – 6.6	5.3 – 6.5	5.9 – 6.9	5.2 – 6.8	
	SD	1.7	1.2	1.6	1.2	1.8	
Vitality	MEAN	3.0	5.9	5.7	6.4	6.2	<0.001
	95% CI	2.4 – 3.6	5.3 – 6.5	5.0 – 6.3	6.0 – 6.9	5.5 – 6.9	
	SD	1.8	1.2	1.7	1.1	1.5	
QOLRAD total	MEAN	3.1	6.1	5.6	6.3	6.0	<0.001
	95% CI	2.6 – 3.7	5.6 – 6.6	5.0 – 6.3	5.9 – 6.8	5.2 – 6.7	
	SD	1.6	1.0	1.6	1.2	1.6	

**Figure 6:**
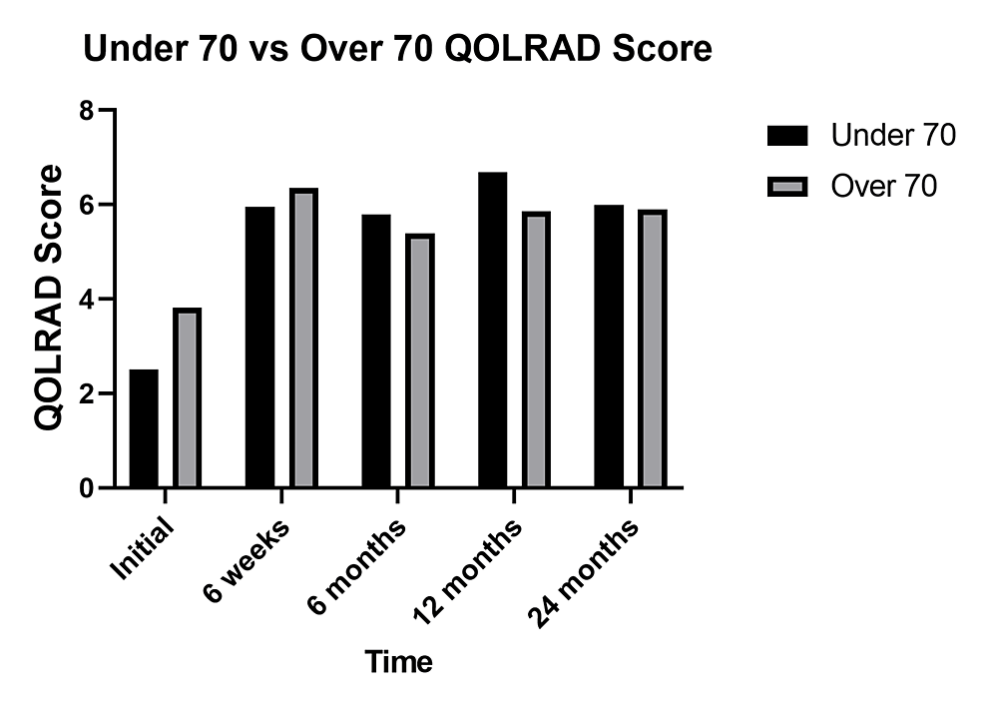
Column bar chart comparison of QOLRAD scores categorised by age Paired comparison of total mean QOLRAD scores in patients above and below 70. Initially QOLRAD score was significantly higher in the over 70 group (3.82 vs 2.50, p=0.037). No significant difference was found in QOLRAD scores between the over 70 and under 70 groups at six weeks (6.36 vs 5.96, p=0.102), six months (5.39 vs 5.80, p=0.990), 12 months (5.86 vs 6.69, p=0.294) and 24 months (5.90 vs 6.00, p=0.685). QOLRAD - Quality of Life in Reflux and Dyspepsia

**Figure 7:**
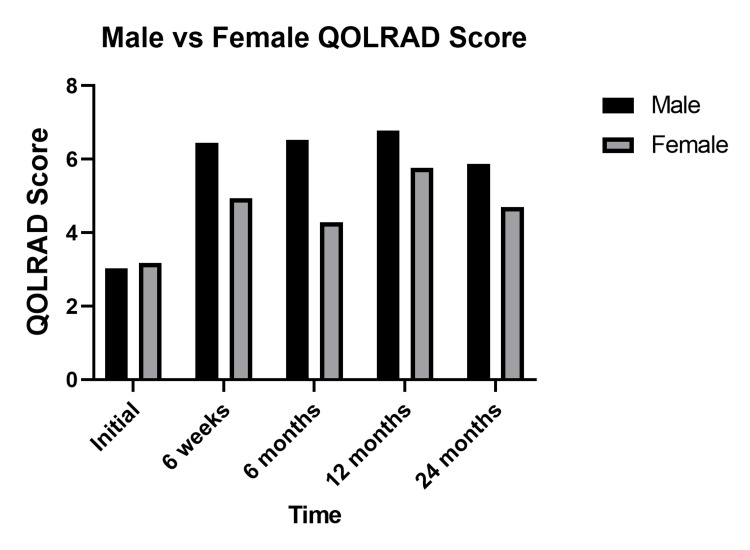
Column bar chart comparison of QOLRAD scores categorized by gender Paired comparison of gender in total mean QOLRAD scores. Postoperatively men had significantly higher QOLRAD scores at six weeks (6.44 vs 4.94, p=0.016), six months (6.52 vs 4.28, p=0.003) and 12 months (6.78 vs 5.77, p=0.043). No significant difference in QOLRAD scores between males and females was observed on initial scoring (3.03 vs 3.18, p=0.644) and at 24 months (5.88 vs 4.69, p=0.225). QOLRAD - Quality of Life in Reflux and Dyspepsia

Multiple Mann-Whitney U tests revealed no statistically significant difference in an overall improvement in QOLRAD scores postoperatively in IOG and DOG when patients were categorized by age above and below 70. The initial QOLRAD score was significantly higher in the over 70 groups in comparison to the under 70 groups (3.82 vs 2.50, p=0.037). When categorized by gender, the QOLRAD scores of men were significantly higher at six weeks, six months, and 12 months postoperatively. Figures 6, 7 present these comparisons in the form of column bar charts.

## Discussion

Of the 56 patients who underwent operative management, there were no mortalities, and four patients (7.1%) required a re-operation for recurrence demonstrating that GHH repair is a safe operation with low recurrence rates and excellent QoL improvement up to two years postoperation. This finding is consistent with Date et al.'s systematic review of QoL post-GHH repair [16]. The QOLRAD tool demonstrated significant improvements in all QoL domains. This QoL improvement was similar to those who underwent immediate and delayed GHH repair.

Age had little impact on postoperative improvement in QoL, a similar finding was made by Sgromo et al. and Houghton et al. when comparing the QoL post-Nissen and Toupet fundoplication with gastroplasty [17,18]. Improvement in QOLRAD scores post-GHH repair in DOG patients indicated in Figure 4 proves that delay in GHH repair does not impact QoL improvement postoperatively.

Nijhuis et al. have conducted a long-term retrospective study on long-term outcomes of conservatively managed GHH in 186 patients, wherein they reported two (1.1%) patients to have required emergency surgery, 13 (7.0%) patients to have required elective surgery and an overall mortality rate of 1.61% [19]. In comparison, in our series of 36 patients who were initially managed conservatively, one (2.8%) required emergency surgery and five (13.9%) underwent elective surgery. There were no mortalities due to GHH recorded in our cohort. No other study in the published literature evaluates the impact GHH has on QoL when conservatively managed.

The DOG patients who did later opt to undergo GHH repair were found to have an increase in their postoperative QOLRAD scores comparable to IOG patients. DOG patients had the lowest initial mean QOLRAD scores that were only marginally lower than IOG (2.72 vs 3.15, p=0.743) and, in turn, had significantly lower initial mean QOLRAD scores than NOG (5.05). This suggests a possible correlation between patient choice to undergo initial operative management and QoL.

The difference in median age between NOG and IOG patients (74.0 vs 69.5) is consistent with Stylopoulos et al.'s finding that the risk of progression to severe symptoms decreases with age (4,6). The decision to operate on GHH patients is complex and dependent on patient comorbid factors as well as patient QoL measures. Careful patient selection is crucial in determining which patients would benefit from the most symptomatic relief and functional gain. In our study, five patients were in the DOG, indicating that 13.9% of patients who were initially managed conservatively went on to require elective surgery due to worsening symptoms. DOG patients had significantly lower initial QOLRAD scores in comparison to the NOG patients (2.72 vs 5.05, p=0.034), demonstrated in Figure 3, suggesting that objectively measured QOLRAD scores, as opposed to the perceived quality of life of the patient, may be used to guide surgeons in selecting patients for operative management.

The inherent limitations of an observational study have precluded the ability to match the groups by their symptomatology, age, and co-morbidities. Hence, the impact of these confounding factors and the corresponding selection bias on our results cannot be examined. Our practice of only using a single QoL tool is counter to The European Association of Endoscopic Surgery recommendation of using QOLRAD in combination with the SF-36 questionnaire in assessing quality of life [20]. As such, we have not determined whether a combination of QOLRAD with SF-36 may be more accurate in selecting patients for elective surgery in the future.

## Conclusions

Repair of GHH results in a significant improvement in QoL. Initially conservatively managed patients with GHH with a low initial QOLRAD score may require GHH repair at a later stage and experience a QoL benefit postoperatively. We believe objectively calculated low QoL may be a more useful tool than subjective symptoms in selecting patients for elective repair of GHH.

## Appendices

QOLRAD

QUESTIONNAIRE FOR PATIENTS WITH UPPER ABDOMINAL

SYMPTOMS

PLEASE READ THIS CAREFULLY BEFORE ANSWERING THE QUESTIONS

On the following pages you will find some questions asking about how you have been feeling DURING THE PAST WEEK because of pain or discomfort in your upper abdomen (the upper abdomen is below the ribs and above the belly button). Please answer all of these questions as honestly as you can.

 For each question, tick the box which best describes how you have been feeling.

1.        How often during the past week have you been FEELING TIRED OR WORN OUT BECAUSE OF PAIN OR DISCOMFORT IN THE UPPER ABDOMEN?

¨           All of the time

¨           Most of the time

¨           Quite a lot of the time

¨           Some of the time

¨           A little of the time

¨           Hardly any of the time  ¨ None of the time

2.        How often during the past week did you AVOID BENDING OVER BECAUSE OF CONCERN OVER PAIN OR DISCOMFORT IN THE UPPER ABDOMEN?

¨           All of the time

¨           Most of the time

¨           Quite a lot of the time

¨           Some of the time

¨           A little of the time

¨           Hardly any of the time  ¨ None of the time

3.        During the past week, how much PAIN OR DISCOMFORT IN THE UPPER ABDOMEN HAVE YOU HAD BECAUSE OF EATING OR DRINKING?

¨           A great deal   ¨ A lot 

¨           A moderate amount

¨           Some

¨           A little

¨           Hardly any  ¨ None at all

4.        How often during the past week have you FELT GENERALLY UNWELL BECAUSE OF PAIN OR DISCOMFORT IN THE UPPER ABDOMEN?

¨           All of the time

¨           Most of the time

¨           Quite a lot of the time

¨           Some of the time

¨           A little of the time

¨           Hardly any of the time  ¨ None of the time

5.        How often during the past week was it NECESSARY TO EAT LESS THAN USUAL BECAUSE OF PAIN OR DISCOMFORT IN THE UPPER ABDOMEN?

¨           All of the time

¨           Most of the time

¨           Quite a lot of the time

¨           Some of the time

¨           A little of the time

¨           Hardly any of the time

¨           None of the time

6.        How often during the past week has PAIN OR DISCOMFORT IN THE UPPER ABDOMEN KEPT YOU FROM DOING THINGS WITH FAMILY OR FRIENDS?

¨           All of the time

¨           Most of the time

¨           Quite a lot of the time

¨           Some of the time

¨           A little of the time

¨           Hardly any of the time  ¨ None of the time

7.        How often during the past week did you have A LACK OF ENERGY BECAUSE OF PAIN OR DISCOMFORT IN THE UPPER ABDOMEN?

¨           All of the time

¨           Most of the time

¨           Quite a lot of the time

¨           Some of the time

¨           A little of the time

¨           Hardly any of the time  ¨ None of the time

8.        How often during the past week have you had DIFFICULTY GETTING A GOOD NIGHT’S SLEEP BECAUSE OF PAIN OR DISCOMFORT IN THE UPPER ABDOMEN?

¨           All of the time

¨           Most of the time

¨           Quite a lot of the time

¨           Some of the time

¨           A little of the time

¨           Hardly any of the time  ¨ None of the time

9.        How often during the past week has PAIN OR DISCOMFORT IN THE UPPER ABDOMEN MADE IT DIFFICULT TO EAT ANY OF THE FOODS OR SNACKS YOU LIKE?

¨           All of the time

¨           Most of the time

¨           Quite a lot of the time

¨           Some of the time

¨           A little of the time

¨           Hardly any of the time  ¨ None of the time

10.     How often during the past week did you FEEL TIRED OR WORN OUT DUE TO LACK OF SLEEP BECAUSE OF PAIN OR DISCOMFORT IN THE UPPER ABDOMEN?

¨           All of the time

¨           Most of the time

¨           Quite a lot of the time

¨           Some of the time

¨           A little of the time

¨           Hardly any of the time  ¨ None of the time

11.     How often during the past week did PAIN OR DISCOMFORT IN THE UPPER ABDOMEN WAKE YOU UP AT NIGHT AND PREVENT YOU FROM FALLING ASLEEP AGAIN?

¨           All of the time

¨           Most of the time

¨           Quite a lot of the time

¨           Some of the time

¨           A little of the time

¨           Hardly any of the time  ¨ None of the time

12.     How often during the past week have you felt DISCOURAGED OR DISTRESSED BECAUSE OF PAIN OR DISCOMFORT IN THE UPPER ABDOMEN?

¨           All of the time

¨           Most of the time

¨           Quite a lot of the time

¨           Some of the time

¨           A little of the time

¨           Hardly any of the time  ¨ None of the time

13.     How often during the past week has PAIN OR DISCOMFORT IN THE UPPER ABDOMEN MADE FOOD SEEM UNAPPEALING TO YOU?

¨           All of the time

¨           Most of the time

¨           Quite a lot of the time

¨           Some of the time

¨           A little of the time

¨           Hardly any of the time  ¨ None of the time

14.     How often during the past week have you FELT FRUSTRATED OR IMPATIENT BECAUSE OF PAIN OR DISCOMFORT IN THE UPPER ABDOMEN?

¨           All of the time

¨           Most of the time

¨           Quite a lot of the time

¨           Some of the time

¨           A little of the time

¨           Hardly any of the time  ¨ None of the time

15.     How often during the past week have you been ANXIOUS OR UPSET BECAUSE OF PAIN OR DISCOMFORT IN THE UPPER ABDOMEN?

¨           All of the time

¨           Most of the time

¨           Quite a lot of the time

¨           Some of the time

¨           A little of the time

¨           Hardly any of the time  ¨ None of the time

16.     During the past week, how much PAIN OR DISCOMFORT IN THE UPPER ABDOMEN HAVE YOU HAD BECAUSE OF HAVING EATEN FOODS OR SNACKS YOU COULD NOT TOLERATE?

¨           A great deal   ¨ A lot 

¨           A moderate amount

¨           Some 

¨           A little

¨           Hardly any  ¨ None at all

17.     How often during the past week have you had ANY WORRIES OR FEARS ABOUT YOUR HEALTH BECAUSE OF PAIN OR DISCOMFORT IN THE UPPER ABDOMEN?

¨           All of the time

¨           Most of the time

¨           Quite a lot of the time

¨           Some of the time

¨           A little of the time

 ¨

18.     How often during the past week did you FAIL TO WAKE UP IN THE MORNING FEELING FRESH AND RESTED BECAUSE OF PAIN OR DISCOMFORT IN THE UPPER ABDOMEN?

¨           All of the time

¨           Most of the time

¨           Quite a lot of the time

¨           Some of the time

¨           A little of the time

¨           Hardly any of the time  ¨ None of the time

19.     How much during the past week has PAIN OR DISCOMFORT IN THE UPPER ABDOMEN MADE YOU FEEL IRRITABLE?

¨           A great deal   ¨ A lot 

¨           A moderate amount

¨           To some extent

¨           A little

¨           Hardly at all  ¨ Not at all

20.     How often during the past week have you had to AVOID CERTAIN FOOD, BEVERAGES OR DRINKS BECAUSE OF PAIN OR DISCOMFORT IN THE UPPER ABDOMEN?

¨           All of the time

¨           Most of the time

¨           Quite a lot of the time

¨           Some of the time

¨           A little of the time

 ¨

21.     How often during the past week did you HAVE TROUBLE GETTING TO SLEEP BECAUSE OF PAIN OR DISCOMFORT IN THE UPPER ABDOMEN?

¨           All of the time

¨           Most of the time

¨           Quite a lot of the time

¨           Some of the time

¨           A little of the time

¨           Hardly any of the time  ¨ None of the time

22.     How often during the past week did you FEEL FRUSTRATED BECAUSE THE EXACT CAUSE OF YOUR SYMPTOMS IS NOT KNOWN AND YOU STILL HAVE SO MUCH PAIN OR DISCOMFORT IN THE UPPER ABDOMEN?

¨           All of the time

¨           Most of the time

¨           Quite a lot of the time

¨           Some of the time

¨           A little of the time

¨           Hardly any of the time  ¨ None of the time

23.     How often during the past week did you have DIFFICULTY SOCIALIZING WITH FAMILY OR FRIENDS BECAUSE OF PAIN OR DISCOMFORT IN THE UPPER ABDOMEN?

¨           All of the time

¨           Most of the time

¨           Quite a lot of the time

¨           Some of the time

¨           A little of the time

 ¨

24.     How often during the past week were you UNABLE TO CARRY OUT YOUR DAILY ACTIVITIES (INCLUDING BOTH WORK OUTSIDE THE HOME AND HOUSE WORK) DUE TO PAIN OR DISCOMFORT IN THE UPPER ABDOMEN?

¨           All of the time

¨           Most of the time

¨           Quite a lot of the time

¨           Some of the time

¨           A little of the time

¨           Hardly any of the time  ¨ None of the time

25.     How often during the past week were you UNABLE TO CARRY OUT YOUR NORMAL PHYSICAL ACTIVITIES (INCLUDING SPORT, LEISURE ACTIVITIES AND MOVING AROUND OUTSIDE THE HOME) DUE TO PAIN OR DISCOMFORT IN THE UPPER ABDOMEN?

¨           All of the time

¨           Most of the time

¨           Quite a lot of the time

¨           Some of the time

¨           A little of the time

¨           Hardly any of the time  ¨ None of the time

PLEASE CHECK THAT YOU HAVE ANSWERED ALL THE QUESTIONS!

THANK YOU FOR YOUR CO-OPERATION.
